# Hydrocarbon generation potential and geochemical characteristics comparison of source rocks in the Southwestern Qaidam basin, China

**DOI:** 10.1038/s41598-025-92846-4

**Published:** 2025-03-10

**Authors:** Yang Zhao, Chenxin Huang, Fenjun Chen, Qingyou Yue

**Affiliations:** 1 College of Petroleum and Natural Gas Engineering, Liaoning Petrochemical University, Fushun, 113000 Liaoning China; 2 Exploration & Production Research Institute of Qinghai Oilfield Company, PetroChina, Dunhuang, 736200 China

**Keywords:** Source rock, Cluster analysis, Geochemical characteristics, Biomarkers, Qaidam basin, Geochemistry, Geology

## Abstract

A primary source rock has developed in the Upper part of the Lower Ganchaigou Formation (E_3_^2^) in the southwestern Qaidam Basin, China. This basin features typical brackish-saline lacustrine deposits, necessitating careful selection of appropriate standards for evaluating hydrocarbon generation potential. The presence of multiple sets of source rocks and complex migration pathways has led to the accumulation of mixed-source oils, complicating the relationship between crude oil and source rocks and the establishment of hydrocarbon migration system. To address these challenge, 113 source rock samples were analyzed using Rock–Eval 6, gas chromatography-mass spectrometry and microphotometer. R-type clustering and principal component analysis were employed to select two out of five biomarker parameters that reflect water salinity and parent material sources for Q-type clustering. The results indicate that the E_3_^2^ source rock exhibits fair hydrocarbon potential and is predominantly composed Type I and Type II_1_ kerogens. It remains in a low-maturity to mature stage, with deposition occurring in environments characterized by either strong reduction and high salinity or relatively weak reduction and low salinity. The oil is derived from nearby source rocks in the Hongshi, Yingxiongling, and Chekrike-Zahazquan depressions. This study provides new insights into source rock evaluation and oil-source relationship analysis.

## Introduction

Given the rapid growth in global energy demand and the gradual depletion of conventional hydrocarbon reserves, exploring new resources has become critically important. This is essential not only for ensuring energy security but also for sustaining economic growth and driving technological innovation. Source rock, which provides the fundamental organic material basis for hydrocarbon generation, play a central role in the processes of hydrocarbon migration, accumulation, and preservation^1–4^. The evaluation of source rock begins with a comprehensive examination of the origin, depositional environment, quantity, type, maturity of organic matter conversion to petroleum^3,5^. The quantity of organic matter in source rock is measured by geochemical and microscopic analysis of rock samples^1,5,6^. In situations where core or cutting samples are scarce, the integration of organic geochemical data with well logging and seismic data can be used to construct mathematical models for predicting the total organic carbon (TOC) content^2,7^. Researchers widely utilize Rock-Eval 6 pyrolysis parameters and microscopic components to identify kerogen types^1,3,5^. For maturity assessment, rock pyrolysis analysis and biomarker compounds are the primary means of application, and some researchers further integrate seismic data, biomarker compounds, and pyrolysis data to expand the scope of maturity prediction^1^. As for determining the source of organic matter and the paleo-depositional environment, the analysis mainly relies on the combination of elemental geochemistry and organic geochemistry^1,5,8^. These analytical methods and techniques have been widely applied in the evaluation of source rock and are becoming increasingly mature. However, due to the diversity of depositional environments and the complexity of geological conditions, the geochemical characteristics of source rock and the properties of the generated fluids are difficult to accurately differentiate, which directly affects the establishment of a predictive model for generation and charge within a petroleum system.

The Qaidam Basin is an important petroliferous basin in western China, recent advancements in source rock research have significantly contributed to breakthroughs in hydrocarbon exploration within the region^9–14^. The discovery of oil has further heightened interest in its exploration potential. During the Tertiary period, the Qaidam Basin experienced a predominantly arid climate, which favored the widespread development of brackish-saline lacustrine deposits. Conventional studies suggest that the quality, scale, and distribution of source rock directly influence the formation and distribution of hydrocarbon^15–18^. High-quality source rock deposited in lacustrine environment with high organic matter content form the basis for large-scale oilfield^19,20^. In line with these principles, an illustrative case is provided by the work of Zhang et al. (2017), after analyzing numerous samples, who concluded that type II_2_and III organic matter with TOC less than 0.5% are ineffective as source rock, thus suggesting the TOC threshold should be adjusted to 0.5% for the western Qaidam Basin^13^. Conversely, other research has found that despite the low organic matter abundance and suboptimal source rock developed in the western Qaidam Basin, they exhibit high hydrocarbon generation potential^21^. Li et al. (2006) proposed that the TOC threshold should be 0.4%, based on the analysis of the relationship between the chloroform bitumen ‘A’ and TOC, as well as pyrolysis parameters^22^. Although the 0.1% difference between the two standards may seem minor, it could significantly affect the evaluation range of source rock, thereby influencing hydrocarbon exploration and the reserves calculation. Therefore, assessing the quality of the lacustrine source rock and determining the appropriate criteria for evaluation become a critical issue in the field of petroleum geology.

The western Qaidam Basin depression features multiple sets of source rocks development, with hydrocarbons from these sources accumulating in specific traps to form mixed-source oil reservoirs. Studies also have shown that different sedimentary microfacies within a single source rock exhibit varying geochemical characteristics. Conversely, similar geochemical characteristics across different stratigraphic series, likely due to comparable depositional environments^23^, further complicate the correlation of oil with its source rocks. The study of mixed-source oils has been a focal point for researchers worldwide. Peters and Moldowan (2001) introduced a laboratory-blend-based computational method to assess the relative contributions of mixed-source oils^24^. However, this method is limited by the difficulty of obtaining pure single-source oil samples in practical applications. Wang et al. (2001) successfully identified mixed-source oils in the Junggar Basin through the analysis of the C_20_, C_21_, and C_23_tricyclic terpanes^25^, which most of the crude oils found in other study area do not completely conform to^26^. Subsequently, Peters et al. (2008) developed a chemometric approach that calculates the contribution ratios of different end-member oils without the need for pure samples^27^, which has since been successfully applied in multiple oilfields^26,28^. Traditionally, predicting hydrocarbon migration pathways has primarily relied on content variations in crude oil maturity, biomarkers, and isotope. However, in mixed-source hydrocarbon systems, the mixed-oils from different source rock types and maturity levels obscures these variations, making it difficult to accurately reflect the true migration pathways^29^.

Analysis reveals that the formation of mixed-source oils is primarily attributed to multiple sets of source rocks and complex migration pathways. Quantitative differentiation of source rocks and their affinities with crude oil will aid in pinpointing the specific origins of mixed-source crude oils, thereby elucidating the hydrocarbon migration pathways and the distribution characteristics of petroleum systems. As machine learning technology continues to evolve, it has found increasing utility in the processing of geochemical data. Specifically, unsupervised learning techniques, such as cluster analysis, have proven effective in categorizing unlabeled data and revealing the latent meanings inherent in geochemical data.

This study conducted a comprehensive analysis of the hydrocarbon generation potential of source rock by collecting a comprehensive set of samples from the main source rock in the southwestern Qaidam basin and integrating them with previous data. Based on the cluster analysis theory, a comparative study was performed to examine the differences and similarities in the geochemical characteristics among the source rocks, which will further address the relationship between mixed-source oils and source rocks when combined with oil samples. The application of cluster analysis techniques can also reveal hydrocarbon migration pathways, which can guide subsequent exploration wells to increase the probability of discovery. This approach benefits researchers worldwide by enabling a more effective evaluation of source rock in basins with similar geological conditions.

## Geological setting

**Fig. 1:**
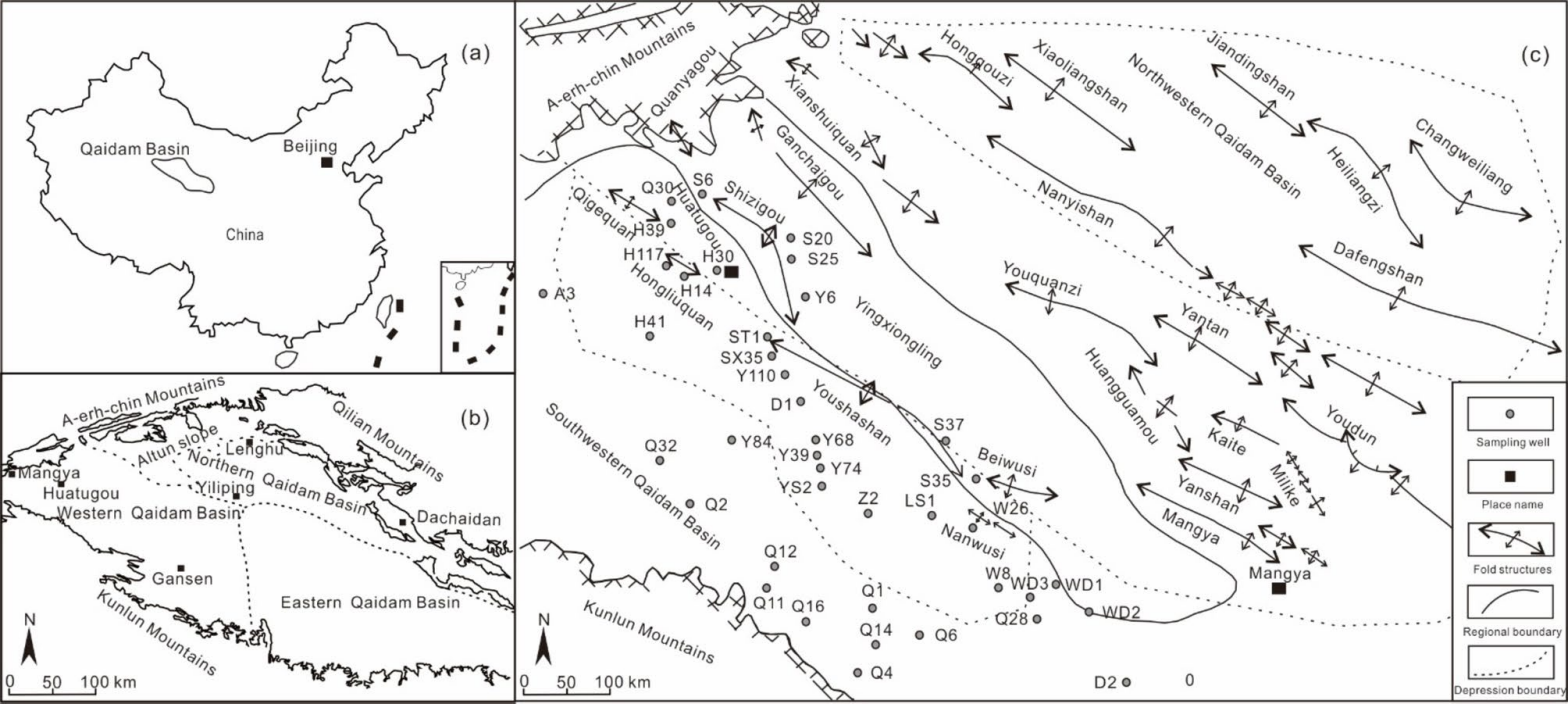
(**a**) Location of the Qaidam Basin. (**b**) The structural pattern of the Qaidam Basin. (**c**) The southwestern extent of the Qaidam Basin and the distribution of folds and source rock sampling wells. Owing to the dense distribution of the sampling wells, only a subset of the wells is annotated.

The Qaidam Basin, one of the three major inland basins in northwest China, is located on the northern margin of the Qinghai-Tibet Plateau. The western Qaidam Basin, covering an area of approximately 27,000 km^2^, lies west of the Niubiliang-Dongchai Mountain line. It is bordered by the A-erh-chin Mountains to the northwest and the Kunlun Mountains to the south. This region includes the Kunbei fault steps, the Mangya depression, and the Dafengshan bulge, with an area of source rocks superimposed over 14,872 km^2^. The estimated total oil and gas resources in this area are 21.93 billion tons, making it the most abundant oil and gas region within the Qaidam Basin. The western Qaidam area is subdivided into the southwestern Qaidam Basin and the northwestern Qaidam, with the Yingxiongling sag serving as the boundary^11^ (Fig. 1).

The study area includes various strata, listed in ascending order as follows: the Lulehe Formation (E_1+2_), the Lower part of the Lower Ganchaigou Formation (E_3_^1^), the Upper part of the Lower Ganchaigou Formation (E_3_^2^), the Upper Ganchaigou Formation (N_1_), the Lower Youshashan Formation (N_2_^1^), the Upper Youshashan Formation (N_2_^2^), the Shizigou Formation (N_2_^3^), and the Qigequan Formation (Q)^13^. Among these, the Upper part of the Lower Ganchaigou Formation (E_3_^2^) is recognized as the primary source rock in the region^30^.

## Materials and methods

A total of 113 source rock samples were carefully collected in the study area. Due to the close spacing of the wells, only a selection of these wells was marked (Fig. 1). Each sample underwent a systematic process. Initially, the samples were finely crushed to pass through a 100 mesh sieve, and 50 g of the finely crushed material were weighed. It was then subjected to extraction using a Soxhlet extractor for 72 h, resulting in a chloroform extract. Next, the extract was precipitated with n-hexane and filtered to obtain the deasphalted fraction. Subsequent separation of the deasphalted components was further achieved using alumina/silica gel chromatography, which led to the isolation of saturated and aromatic hydrocarbon fractions^31,32^. The saturated hydrocarbon fractions were identified through gas chromatography-mass spectrometry (GC-MS) (21 samples). Additionally, the source rock pyrolysis parameters and TOC were thoroughly analyzed using the advanced Rock-Eval 6 rock pyrolysis instrument (63 samples). The vitrinite reflectance (R_o_) of the samples was accurately determined using a microscope combined with a microphotometer (29 samples ). Utilizing a substantial amount of accumulated crude oil analysis data from the oilfield, this study employed GC-MS analysis data from 23 existing crude oil samples, without collecting new samples.

Prior to conducting a comparative analysis of the geochemical characteristics of source rocks through cluster analysis, the data must first be standardized to ensure consistency and comparability. Subsequently, the Squared Euclidean Distance is computed to quantify the dissimilarities between samples. The detailed procedural steps are outlined as follows:

Standardization was achieved by subtracting the minimum value from the each datum and dividing by the range, scaling the transformed values within a 0 to 1 interval, denoted as:

1 $$x^*\:=\:\frac{x-{x}_{min}}{{x}_{max}-{x}_{min}}$$

where x* represents the standardized data, x denotes the data before standardization, x_max_ is the maximum value of the data before standardization, and x_min_ is the minimum value of the data before standardization.

Following the standardization, the next step involves calculating the Squared Euclidean Distance (SED), denoted as:

2 $$\:SED=\sum_{i=1}^{n}{\left({X}_{i}-{Y}_{i}\right)}^{2}$$

where SED stands for the Squared Euclidean Distance, X_i_ and Y_i_ represent the values of the *i*-th feature for two samples, respectively.

## Results

**Fig. 2:**
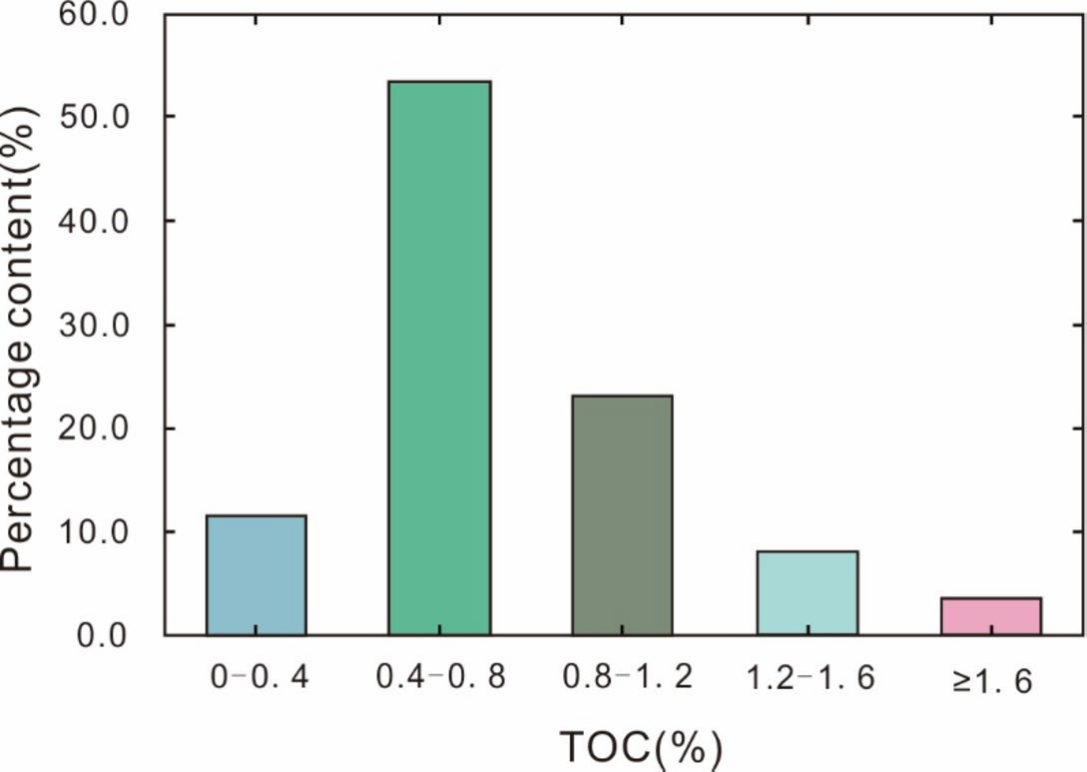
Distribution of TOC obtained by Rock-Eval 6 for the source rock samples from E_3_^2^ in the southwestern Qaidam Basin, China. This chart illustrates the percentage content of different TOC (Total Organic Carbon) ranges. The majority of samples (53.6%) have a TOC content between 0.4% and 0.8%, followed by the range of 0.8–1.2%. Samples with TOC content below 0.4% or above 1.6% are relatively less common.

**Fig. 3:**
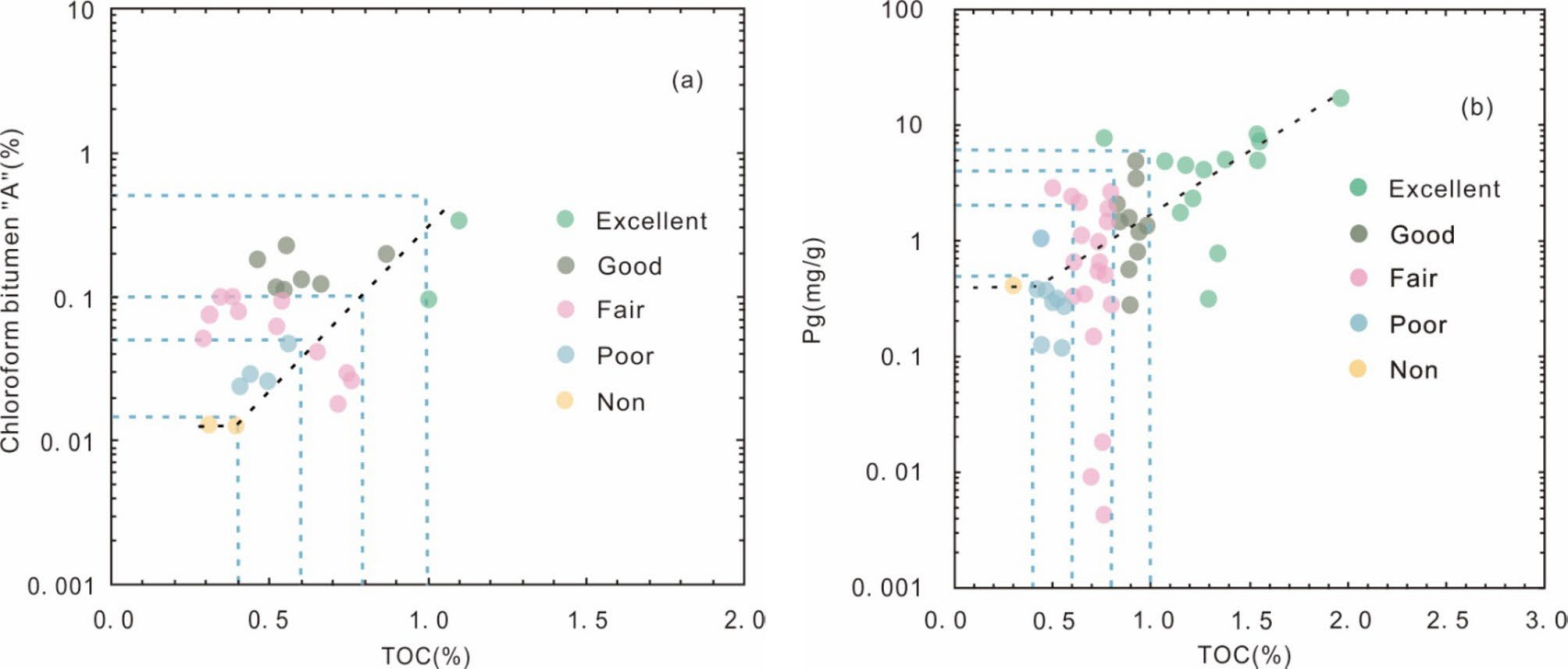
Plots of (**a**) Chloroform bitumen”A” vs TOC, (**b**) P_g_ vs TOC for the source rock samples from E_3_^2 ^in the southwestern Qaidam Basin, China. The black lines denote trend lines, with their intersection marking the trend’s turning point. As proposed by Lu et al. (2012), this turning point represents the lower TOC threshold for effective source rock^33^. When the TOC content surpasses 0.4%, both chloroform bitumen‘A’ and hydrocarbon generation potential (Pg) exhibit a positive correlation with TOC, with the exception of a few outliers, indicating that the threshold TOC value for the source rock is 0.4%.

**Fig. 4:**
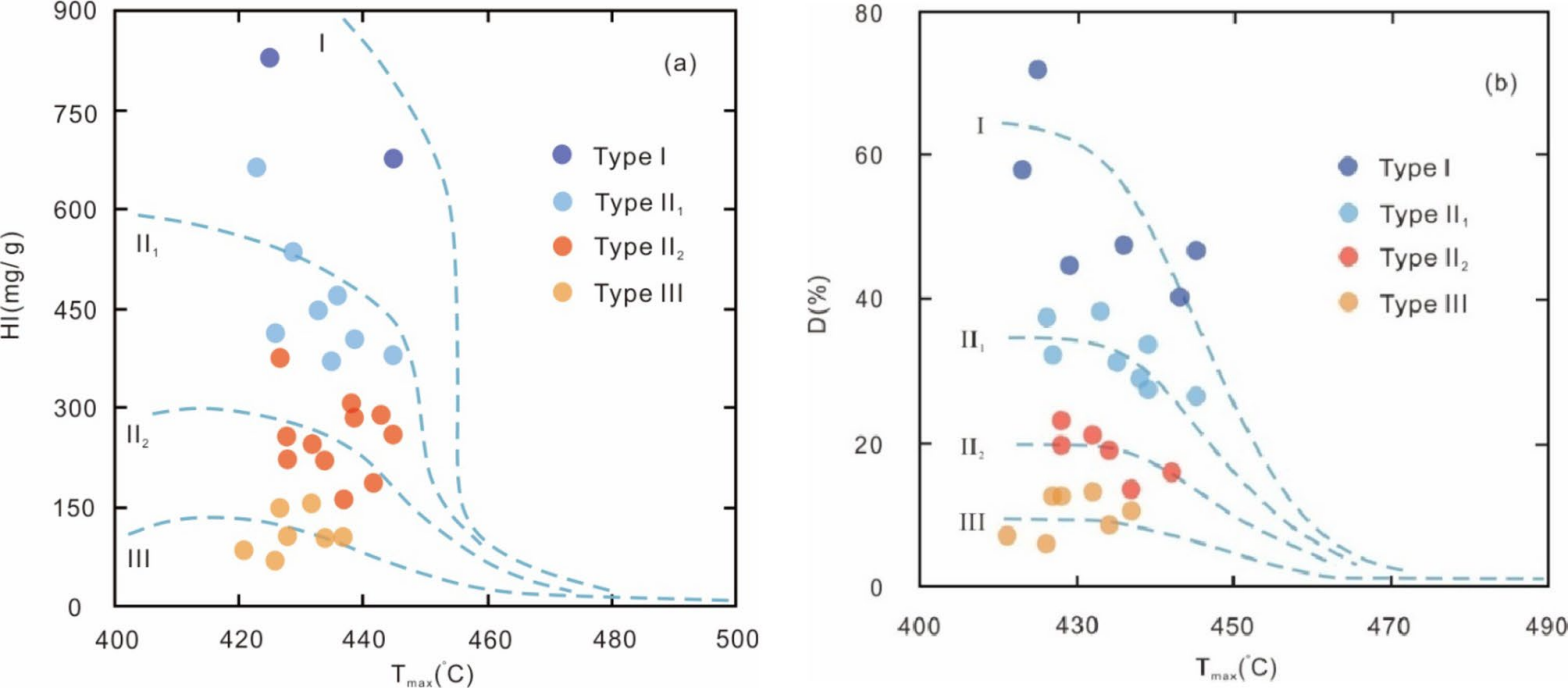
Plots of (**a**) Hydrogen index (HI) vs. Maximum pyrolysis temperature (T_max_), (**b**) Degradation rate (D) vs. Maximum pyrolysis temperature (T_max_) for the source rock samples from E_3_^2^ in the southwestern Qaidam Basin, China. The plots show the distribution of kerogen types in the source rock samples.

Hydrocarbon group composition analysis reveals average values of 49.35% for saturated hydrocarbons, 22.07% for aromatic hydrocarbons, 18.07% for resins, and 10.51% for asphaltenes. The TOC content of the source rock samples varies widely, ranging from 0.23–2.86%, with a mean of 0.77%. The highest proportion of samples (53.6%) exhibits TOC content ranging from 0.4–0.8%, while 88.4% of the samples have TOC content greater than 0.4% (Fig. 2). Chloroform asphaltene ‘A’ content ranges from 0.013 to 0.339%, with an average of 0.096% (Fig. 3a). The hydrocarbon generation potential (Pg) values fluctuate between 0.004 mg/g and 17.86 mg/g, with an average of 2.28 mg/g (Fig. 3b). Hydrogen index (HI) values range from 15.72 mg/g to 826.52 mg/g, averaging 189.53 mg/g. Degradation rate (D) values span from 1.78 to 84.73%, with an average of 18.16%. The maximum pyrolysis temperature (T_max_) values span from 366 °C to 445 °C, with an average of 431 °C (Fig. 4).

**Fig. 5:**
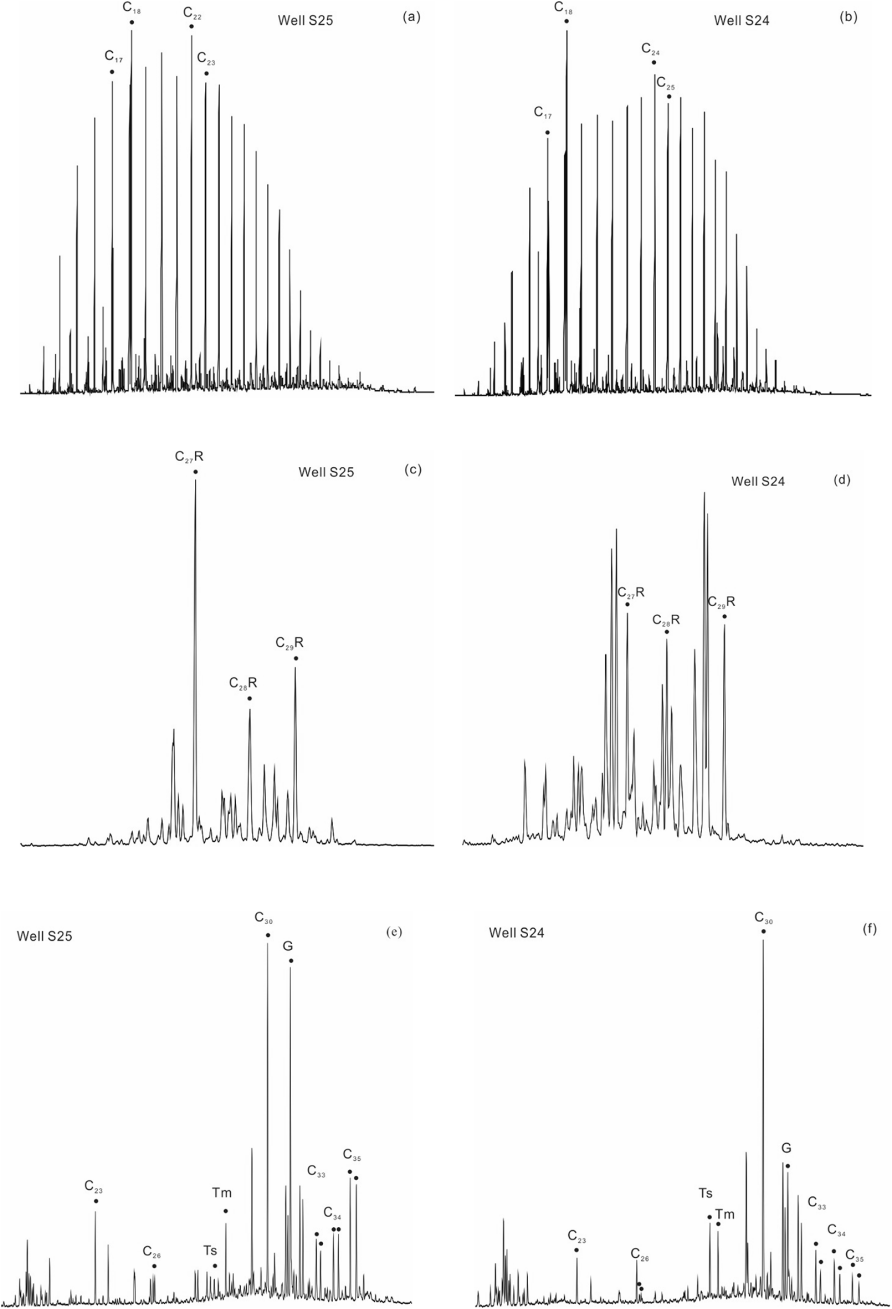
(**a**) and (**b**): representative mass chromatograms of m /z 85 for the source rock from E_3_^2^ in the southwestern Qaidam Basin, China. **a** Bimodal distribution, with major peaks at carbon C_18_ and C_22_. **b** Bimodal distribution, with major peaks at carbon C_18_ and C_24_. (**a**) and (**b**) depict the presence of organic matter originating from both higher plants and aquatic sources. (**c**) and (**d**): representative mass chromatograms of m /z 217 for the source rock from E_3_^2^ in the southwestern Qaidam Basin, China. **c** The content of C_27_ ααR is significantly higher than that of C_29_ ααR sterane. **d** The C_27_ ααR content slightly exceeds that of the C_29_ ααR sterane. (**c**) and (**d**) illustrate the presence of organic matter derived from both higher plants and aquatic sources, with a higher proportion originating from aquatic organisms. (**e**) and (**f**): representative mass chromatograms of m /z 191 for the source rock from E_3_^2^ in the southwestern Qaidam Basin, China. **e** C_23_ stands out as the primary peak, while C_26_ tricyclic terpenes exhibit a bimodal distribution. Additionally, there is a notable presence of gammacerane, with hopane content following the order C_33_ < C_34_ < C_35_, indicative of a high salinity environment. **f** The C_23_ primary peak is not prominent, while C_26_ exhibits a bimodal distribution. Gammacerane content is relatively low, with hopane content following the order C_33_ > C_34_ > C_35_, suggesting a low salinity environment.

The n-alkane carbon numbers in the study samples range from C_12_ to C_38_ (Fig. 5a, b; Table 1). The phytane series is notably abundant, with the Pr/Ph ratio ranging from 0.14 to 0.57 and an average of 0.33, which is significantly less than 1.0. The Pr/nC_17_ ratio varies between 0.09 and 1.98, averaging 0.53, while the Ph/nC_18_ ranges from 0.13 to 6.75, with an average of 2.03. The Pr + Ph/(C_17_ + C_18_) ratio varies between 0.11 and 4.40, averaging 1.29. The odd-even predominance (OEP) ranges from 0.58 to 1.14, and the carbon preference index (CPI) spans from 0.70 to 3.35. In particular, samples with OEP and CPI values less than 1 also exhibit Ph/nC_17_ and Ph/nC_18_ ratios that surpass 0.5 (Table 1).

**Fig. 6:**
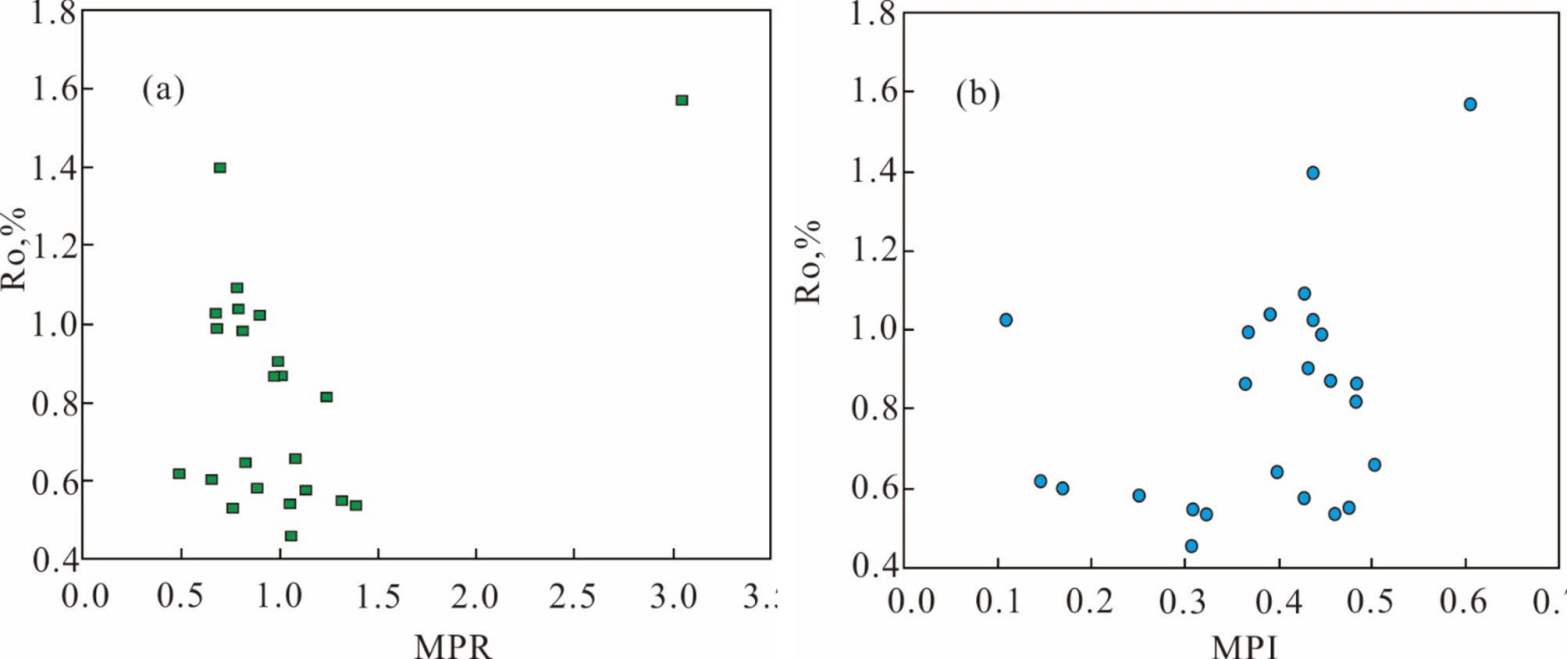
Plots of (**a**) Ro vs. MPR, (**b**) Ro vs. MPI for the source rock samples from E_3_^2^ in the southwestern Qaidam Basin, China. (**a**) With the exception of the point on the right side, generally decreases as MPR increases. (**b**) The correlation between MPI and Ro is not strongly evident, and in some cases, there is even a negative correlation. Where: Ro: vitrinite reflectance. MPI: methylphenanthrene index. MPR: methylphenanthrene ratio. MPR = (3-MP + 2-MP)/(9-MP + 1-MP); MPI = 1.5 × (3-MP + 2-MP)/(*P* + 1-MP + 9-MP); 3-MP: 3-Methylphenanthrene, 2-MP: 2-Methylphenanthrene, 9-MP: 9-Methylphenanthrene, 1-MP: 1-Methylphenanthrene, P: Phenanthrene.

The content of C_28_ sterane is relatively high, and the C_27_ ααR/C_29_ ααR sterane ratio exceeds 1.0 (Fig. 5c, d). Additionally, the Ts/Tm ratio ranges from 0.1 to 3.32, with an average value of 0.72, while C_29_ Ts/C_29_ hopane ratio also ranges from 0.1 to 3.32, with an average value of 0.79. Two distribution patterns for C_34_ and C_35_ hopanes are observed: C_34_/C_35_ < 1 in one pattern (Fig. 5e) and C_34_/C_35_ > 1 (Fig. 5f) in the other. The R_o_ values vary from 0.45 to 0.91%, with an average of 0.65% (Fig. 6a, b).

**Table 1:** Analytical data of n-alkanes and isoprenoid alkanes in source rock from the E_3_^2^ in the Southwestern Qaidam basin, China. Pr/Ph: the ratio of pristane to phytane. Pr/nC_17_: the ratio of pristane to n-heptadecane. Ph/nC_18_: the ratio of phytane to n-octadecane. Pr + Ph/(C_17_ + C_18_): the ratio of the sum of pristane and phytane to the sum of n-heptadecane and n-octadecane. CPI: the carbon preference index. OEP: the Odd-Even predominance. carbon number: the number of carbon atoms in n-alkane molecules.

Well name	Pr/Ph	Pr/nC_17_	Ph/nC_18_	Pr + Ph/(C_17_ + C_18_)	CPI	OEP	Carbon number
A2	0.42	0.34	1.22	0.69	2.18	1.13	C_12−37_
JS1	0.14	1.27	6.75	4.4	0.82	0.67	C_14−35_
QX6-5	0.23	0.37	1.45	0.94	0.97	0.86	C_12−37_
Q4	0.51	0.44	1.65	0.85	3.35	1.14	C_13−35_
W8	0.53	0.35	0.98	0.60	1.98	1.13	C_12−35_
LS1-1	0.40	0.26	0.88	0.52	1.17	1.13	C_14−37_
LS1-2	0.57	0.77	1.65	1.17	1.26	0.98	C_14−36_
LS1-3	0.37	0.41	1.24	0.80	1.13	0.92	C_14−38_
LS1-4	0.29	0.28	0.98	0.63	1.15	1.04	C_15−38_
LS1-5	0.66	0.09	0.13	0.11	1.04	0.93	C_13−38_
LS1-6	0.42	0.16	0.30	0.24	0.99	1.01	C_12−38_
S25-1	0.24	0.39	1.20	0.85	0.97	0.84	C_14−38_
S25-2	0.25	0.41	1.38	0.94	0.90	0.79	C_13−38_
S31	0.36	1.98	5.22	3.63	0.93	0.85	C_14−35_
SS24	0.29	0.68	2.06	1.42	0.89	0.86	C_14−37_
YH101-1	0.16	0.53	3.97	2.08	0.81	0.68	C_13−37_
YH101-2	0.13	0.52	3.24	2.01	0.87	0.88	C_12−38_
YH101-3	0.17	0.50	3.00	1.73	0.70	0.68	C_13−38_
YH101-4	0.16	0.48	2.58	1.61	0.76	0.70	C_13−38_
YH101-5	0.26	0.49	1.66	1.11	1.32	0.58	C_13−38_
YH101-6	0.57	0.50	1.04	0.75	3.28	1.04	C_12−37_

## Discussion

### Organic matter abundance

The abundance of organic matter is a key determinant of hydrocarbon potential. Pyrolysis parameters, such as the Pg, provide critical metrics for assessing the quantity of organic matter in source rock. The types of organic matter found in terrestrial basins are highly complex and can vary significantly. This variation leads to divergent evaluations among researchers, influenced by factors such as sedimentary basin conditions, stages of hydrocarbon generation, and evolutionary characteristics. These differences underscore the importance of recognizing the distinct geochemical characteristics of source rock across different study areas and the need for region-specific evaluation standards.

During the Tertiary period, the Qaidam Basin experienced arid climatic conditions, leading to predominantly brackish to saline lacustrine sedimentation. As a result, Tertiary source rock in the western Qaidam Basin exhibit high salinity and carbonate content^34^. These geochemical features are consistent with those of saline lacustrine source rock formed under arid climatic conditions, exhibiting distinct depositional and compositional attributes. Therefore, the application of conventional evaluation standards for lacustrine source rock may not be appropriate.

Lu et al. (2012) suggested that when the TOC content is low, the volume of hydrocarbons generated by the source rock is minimal. Under such conditions, the generated hydrocarbons are predominantly retained within the source rock through mechanisms such as adsorption, dissolution, or pore entrapment, thereby inhibiting their expulsion. As the TOC content increases, the volume of generated hydrocarbons also rises. Once the hydrocarbon volume exceeds the retention capacity of the source rock, the excess hydrocarbons are expelled in significant quantities. This process results in an inflection point on the curve correlating TOC content with hydrocarbon expulsion efficiency. This inflection point serves as a critical criterion for establishing the lower TOC threshold for effective source rock^33^.

Li et al. (2006) and Zhang (2023) identified the adsorption saturation point at TOC = 0.4% through a positive correlation between TOC and Pg in Tertiary source rocks of the southwestern Qaidam Basin^22,35^. The TOC data from the E_3_^2^ source rock indicate that when TOC exceeds 0.4%, both chloroform bitumen ‘A’ and Pg show a positive correlation with TOC, suggesting that the threshold TOC value for the source rock in the study area is 0.4% (Fig. 3a, b). Based on this result, the evaluation criteria for organic matter abundance established by Li et al. (2006) were applied to assess the hydrocarbon potential of the E_3_^2^source rock samples. The standars are as follows: excellent source rock have TOC greater than 1.0%, good source rock range from 0.8 to 1.0%, fair source rock are between 0.6% and 0.8%, poor source rock fall between 0.4% and 0.6%, and non-source rock have TOC less than 0.4%^22^. TOC values from the E_3_^2^ source rock range from 0.30 to 2.56%, with an average of 0.77%. More than 80% of the samples are classified fair or better, with 32% achieving a rating of good or higher.

In conventional research, Rock-Eval 6 has been widely applied to simulate the thermal cracking of organic matter to generate hydrocarbons and to estimate their hydrocarbon generation potential^36^. However, the thermal vaporization stage of the standard Rock-Eval^®^ Basic/Bulk-RockTM method initiates at a relatively high temperature (300 °C), which can result in the loss of a portion of the remaining free or sorbed hydrocarbons (Rock-Eval 6 S_1_ peak). Consequently, the classical S_1_peak, which quantifies free hydrocarbons, may be underestimated^37^. In recent years, modified thermal vaporization and pyrolysis programs have emerged as critical tool for evaluating unconventional shale resource. A specific Rock-Eval^®^Shale PlayTM method, developed by IFPEN (France), has been introduced to more accurately assess free or sorbed hydrocarbons in unconventional plays^37–40^. This method quantifies the potentially recoverable oil in rock samples using a modified oil saturation index (OSI = Sh0 + Sh1 peaks ×100/TOC) based on the data^37^. However, due to limitations in technological capabilities and experiential knowledge, the novel approach was not adopted in this study. This may represent a potential limitation of the research.

The Pg (S_1_ + S_2_) values range between 0.004 mg/g and 17.86 mg/g, with an average of 2.28 mg/g. Notably, 33% of the samples exceed the threshold for a fair level assessment. Based on the TOC and Pg evaluation results, the E_3_^2^ source rock demonstrates fair hydrocarbon generation potential.

### Organic matter type

The hydrocarbon generation potential in organic matter is closely related to the diverse types of organic compounds. Methods for determining kerogen types include elemental analysis, microscopic component analysis, rock pyrolysis, and saturated hydrocarbon composition analysis^41^. The classification of kerogen types is a human- defined categorization, and scholars hold diverse perspectives on the classification. Tissot and Durand (1974) initially proposed a three-fold classification^42^, which was later expanded by Yang et al. (1981)^43^and Welte and Tissot (1984) into a four-fold classification^44^. Huang et al. (1984) further refined the classification of kerogen types, suggesting a five-fold system^9^. Many researchers have adopted the four-category classification method for kerogen typing. Extensive studies have been conducted on the organic matter types of the E_3_^2^ source rock in the western Qaidam Basin. Based on microscopic identification and elemental composition analysis of kerogen, Peng et al. (2005) determined that the kerogen types are predominantly a mixture of Type I and Type II_1_, with a higher proportion of Type I^34^. Similarly, Zhang et al. (2017) utilized rock pyrolysis and kerogen elemental analysis to conclude that most samples are classified as Type I and Type II_1_, with a minority being Type II_2_ and Type III. Hao (2023) analyzed the relative content of maceral groups and found that the proportions of inertinite and vitrinite are relatively low, while the combined content of liptinite and sapropelinite is notably high, typically exceeding 70%. These results further confirm that the organic matter is primarily Type I and Type II_1_^45^.

The pyrolysis analysis of the E_3_^2^ samples in this study reveals a distribution of all organic matter types, ranging from type I to type III. Among these, Type I and Type II_1_ constitute 51.7% of the total, while Type II_2_ and Type III account for the remaining 48.3% (Fig. 4a, b). The hydrogen index (HI) of Type I kerogen ranges from 676.2 mg/g to 876.5 mg/g, with the degradation rate (D) ranging from 40.1 to 71.9%. For Type II_1_ kerogen, the HI varies between 366.8 mg/g and 661.1 mg/g, and the D ranges from 26.5 to 38.4%. Type II_2_ kerogen exhibits an HI between 158.5 mg/g and 373.3 mg/g, with the D also ranging from 26.5 to 38.4%. In contrast, Type III kerogen has a significantly lower HI, ranging from 69.0 mg/g to 153.1 mg/g, and the D ranges from 6.2 to 10.9%. The HI and D values exhibit clear and distinct differences across the various types of kerogen. Additionally, the average content of saturated hydrocarbons in the sample is 49.35%. Based on these indicators, the kerogen type of the E_3_^2^ source rock is primarily identified as Type I and Type II_1_.

### Organic matter source and depositional environments

Research has identified distinct sources for n-alkanes based on carbon numbers. Specifically, n-alkanes with a carbon numbers below C_21_ are primarily attributed to lower aquatic organisms, whereas those with carbon numbers greater than C_21_are mainly derived from higher plants^45^. Zhang et al. (2019) performed microscopic analysis of organic debris from the E_3_^2^ source rock in the western Qaidam Basin and identified the presence of *Botryococcus*, a green alga from the Chlorophyta phylum, and acritarchs^46^. Hao et al. (2023) conducted a study on E_3_^2^ source rock samples from the Yingxiongling area and found that the ratio of nC_21_^−^/nC_22_^+^ is less than 1, and the relative abundances of C_27_, C_28_, and C_29_steranes exhibit an ‘L’ or inverted ‘L’ pattern. This indicates that the organic matter is primarily derived from plankton and bacteria^47^.

The n-alkane carbon numbers of the E_3_^2^ source rock samples in this study range from C_12_ to C_38_ (Table 1) and exhibit a bimodal distribution (Fig. 5a, b). The primary biomarkers for aquatic algae and higher plants are C_27_ and C_29_sterol, respectively^48^. Given that sterane originates from sterols, they can serve as an indicator of the source of the parent material. The sterane distribution characteristics in the E_3_^2^ samples are notably similar, with C_27_ ααR sterane being predominant. Additionally, the C_27_ ααR/C_29_ ααR sterane ratio exceeds 1.0. This pattern suggests that the parent materials originate from both lower aquatic organisms and higher plants, with a relatively greater contribution from lower algae. These findings are consistent with previous studies.

The phytane series, which is primarily composed of the isoprenoid alkanes found in chloroform asphalt ‘A’, is considered a crucial indicator for discerning the redox conditions of the sedimentary environment in source rock. The distribution and composition traits of this series provide valuable insights into the prevailing oxidative state during deposition.Lower values Pr/Ph, Pr/nC_17_, Ph/nC_18_, and Pr + Ph/(C_17_ + C_18_) ratios typically signify reduced depositional environment, while higher ratios indicate more oxidized conditions^49^. By examining the compositional attributes of both the phytane and n-alkane series within source rock, it is possible to ascertain the redox characteristics of the sedimentary environment. Hopanes are widely distributed in sediments and are recognized as biomarkers indicative of bacterial origin^50^. The distribution characteristics and concentrations of hopanes vary significantly across different depositional environments, reflecting changes in bacterial activity. Gammacerane is a biomarkers recognized for its utility in indicating paleosalinity^48^. Typically, source rock formed in high-salinity depositional environments exhibit higher abundance of gammacerane, while those formed in low-salinity environments are characterized by lower content. Tricyclic terpenes, biomarkers with carbon numbers ranging from C_19_ to C_26_, provide insights into the salinity of depositional environments^51^. Zhang et al. (2012) conducted analyses on samples from the Wunan–Lücaotan area in the western Qaidam Basin, revealing that the Pr/Ph ratios range from 0.18 to 0.89. These values are indicative of a highly reducing environment characteristic of a saline lake^52^. Further supporting this interpretation, Zhang et al. (2019) identified elevated concentrations of phytane, C_28_ steranes, gammacerane, and C_35_hopanes in the source rock. These biomarkers collectively provide strong evidence for a saline depositional environment. The consistency of these results underscores the prevalence of saline lacustrine conditions in the western Qaidam Basin during the depositional period^13^.

In the case of the E_3_^2^ source rock, a prominent presence of the phytane series is evident, with an average Pr/Ph value of 0.33 (Table 1). This value highlights the sedimentary characteristics of a reducing environment. The Tertiary sedimentary period was characterized by predominantly brackish to saline depositional environments^14^. However, periodic fluctuations in lake basin depth and salinity influenced the geochemical features of the source rock. Biomarkers, including hopanes, gammacerane, and tricyclic terpanes provide evidence for two distinct depositional environments. The first environment is marked by a hopane content sequence C_33_ < C_34_ < C_35_, exhibiting a distinctive ‘lifting tail’ trend (Fig. 5e). This pattern is associated with a high gammacerane content, with gammacerane/C_30_ hopane ratios predominantly ranging from 0.60 to 1.20. The samples from this environment also exhibit an inverted ‘V’-shaped distribution, with C_23_ as the main peak carbon and a bimodal distribution of C_26_ tricyclic terpenes, indicative of a depositional environment characterized by strong reduction and high salinity. In contrast, the second environment is indicated by a hopane content in the order C_33_ > C_34_ > C_35_, without the ‘lifted tail’ feature (Fig. 5f). Samples from this setting have low gammacerane content, with gammacerane/C_30_ hopane ratios mostly below 0.6. Furthermore, these samples display low content of C_21_ tricyclic terpenes and lack a prominent C_23_ predominance, suggesting a depositional environment with relatively weak reducing conditions and low salinity.

The Upper part of the Lower Ganchaigou Formation in the Qaidam Basin is characterized by the development of dark shales, overlain by gray to gray-black dolomitic limestones and calcareous mudstones interbedded with multiple salt layers. Mineralogical analysis indicates an average carbonate content of 36.07%, felsic minerals averaging 35.71%, and clay minerals comprising approximately 23.14%. Notably, the presence of pyrite and gypsum further enriches the mineral assemblage, suggesting a dual sedimentary origin characterized by both terrigenous clastic input and endogenic chemical precipitation^53^. Integrating these findings with the geological evolution of the region, the elevated carbonate content and the presence of pyrite provide compelling evidence for a saline, anoxic depositional environment.

### Thermal maturity

The maturity of organic matter in source rock is a critical factor in determining the hydrocarbon generation stage. Ro is highly reliable and is commonly used as the standard parameter for assessing maturity. In the E_3_^2^ samples, R_o_ serves as the primary choice for evaluating maturity. Additionally, existing studies have shown that T_max_can also be an indicator of maturity^54^. Hao (2023) analyzed the organic matter maturity of samples from the Yingxiongling area, revealing that the R_o_ range from 0.68 to 0.85%, with an average of 0.63%^45^. In a complementary study, Zhang et al. (2023) examined 31 samples and documented Ro spanning from 0.46 to 1.28%, with an average of 0.75%^35^. Collectively, these data suggest that the organic matter in the region is within the low-maturity to mature stage of thermal evolution. In this study, the assessment of maturity involved the incorporation of both R_o_ and T_max_ parameters. The results indicate that R_o_ values range from 0.45 to 0.91% (Table 2), with an average of 0.65%, T_max_ values span from 366 °C to 445 °C, averaging 431 °C (Fig. 4). These data suggest that, with the exception of a few samples exhibiting immaturity, the E_3_^2^ samples generally display low-mature to mature thermal maturity.

The presence of aromatic hydrocarbon fractions significantly influences the composition of soluble organic matter within source rock. Compared to saturated hydrocarbon fractions, their geochemical composition is more complex. These fractions not only include conventional polycyclic aromatic hydrocarbons such as naphthalene, phenanthrene, and chrysene but can also exhibit diverse biomarkers with varying degrees of aromatization. Radke and Welte (1981) introduced the methylphenanthrene index (MPI) and established a quantitative relationship between MPI and Ro as an indicator of maturity^55^. However, subsequent research revealed that the MPI is influenced not only by the maturity of the organic matter, but also by other factors such as the depositional environment and the type of organic matter^56,57^.

During an investigation along the northern margin of the Qaidam Basin, Chen et al. (2010) discovered complex correlations between MPI and Ro^58^. In the early stages of organic matter maturation, MPI and Ro demonstrated a positive correlation. However, in the late stages of maturity, an initial negative correlation was observed. This phenomenon presents challenges for accurately assessing crude oil maturity solely based on MPI. Researchers also identified the methylphenanthrene ratio (MPR) as a valuable alternative, which displayed a robust correlation with Ro and offered high predictive accuracy. The analysis of the E_3_^2^ source rock samples reveals intriguing patterns. Ro demonstrates a negative correlation with both MPI and MPR in this study, underscoring the need for caution when employing MPR and MPI to infer the maturity of organic matter in source rock (Fig. 6a, b).

This observation further suggests that the source rock has experienced varying degrees of thermal alteration or microbial degradation. Consequently, the assessment of maturity in this study primarily relies on Ro and Tmax, with OEP and CPI values serving as supplementary reference.

**Table 2:** R_o_ values of E_3_^2^ source rock samples from typical wells in the Southwestern Qaidam basin, China, measured using a microscope-microphotometer system.

Well Name	Area	Lithology	Sampling depth(m)	*R*_o_(%)
H30	Hongliiuquan	Grey mudstone	3300.00	0.46
QD1	Qigequan	Grey mudstone	3200.00	0.53
QD 2	Qigequan	Grey mudstone	3163.00	0.45
QD 3	Qigequan	Grey mudstone	3251.00	0.49
Q1	Chekrike	Grey mudstone	1650.00	0.61
S20	Shizigou	Grey mudstone	2936.00	0.69
S23	Shizigou	Dark gray mudstone	2202.00	0.73
S24	Shizigou	Gray-green mudstone	4200.00	0.86
S27	Shizigou	Grey mudstone	4184.00	0.54
S30	Shizigou	Grey mudstone	3225.77	0.59
W8	Wunan	Grey mudstone	2630.00	0.45
Y14	Youquanzi	Grey mudstone	3000.00	0.91
Y6	Youquanzi	Grey mudstone	4001.00	0.83
Y110	Yuejin	Gray calcareous mudstone	3772.35	0.74
Y146	Yuejin	Grey mudstone	3350.00	0.62

### Comparison of geochemical characteristics

Multiple stratigraphic units of source rock have been identified in the southwestern Qaidam Basin, highlighting accurate determination of their geochemical characteristics. To effectively discern subtle differences among diverse source rock, a sophisticated approach is essential. Cluster analysis, an unsupervised learning technique, has proven to be an effective solution. This method organizes data into distinct groups based on their similarities, primarily using Euclidean distance to measure the relational proximity between samples. By grouping samples with significant similarities into discrete clusters, it enables systematic classification of the entire dataset into meaningful categories.

In this study, five combinations of biomarker parameters were selected to characterize the geochemical properties of source rock (Table 3). The ratios of C_23_/C_21_ tricyclic terpenes and gammacerane/C_31_ homohopane, along with the relative content of pregnane, were used as indicators of the sedimentary water salinity. Meanwhile, the C_24_ tetracyclic terpenes /C_26_ tricyclic terpenes ratio and the C_27_ ααR/C_29_ ααR sterane ratio reflect the source of the parent material. To refine the clustering parameters, R-type clustering was applied to evaluate the relationships among variables, identifying the relative content of pregnane and the C_27_ ααR/C_29_ ααR sterane ratio as parameters for Q-type clustering. Principal component analysis (PCA) of these biomarker combinations showed that the cumulative variance contribution rate (CVCR) of pregnane content and the C_27_ ααR/C_29_ ααR sterane ratio reached 84.3% (Table 4), exceeding the 80% threshold for significant principal components. This underscores their dominant role in shaping dataset variability.

While incorporating Gar/C_31_H could raise the CVCR to 92.689%, it risks increasing model complexity. Thus, Gar/C_31_H was excluded from the PCA.

The C_27_ ααR/ C_29_ ααR sterane ratio ranges from 0.512 to 1.723, averaging 0.978. The relative content of pregnane varies more widely, from 0.308 to 13.052, with an average of 4.649. These datasets display substantial variability. The calculation of the Squared Euclidean distance is considerably influenced by the pregnane content, reducing the contribution of the C_27_ ααR/ C_29_ ααR sterane on the clustering model. Consequently, the normalization of these parameters was necessary to ensure balanced representation in the analysis.

**Fig. 7:**
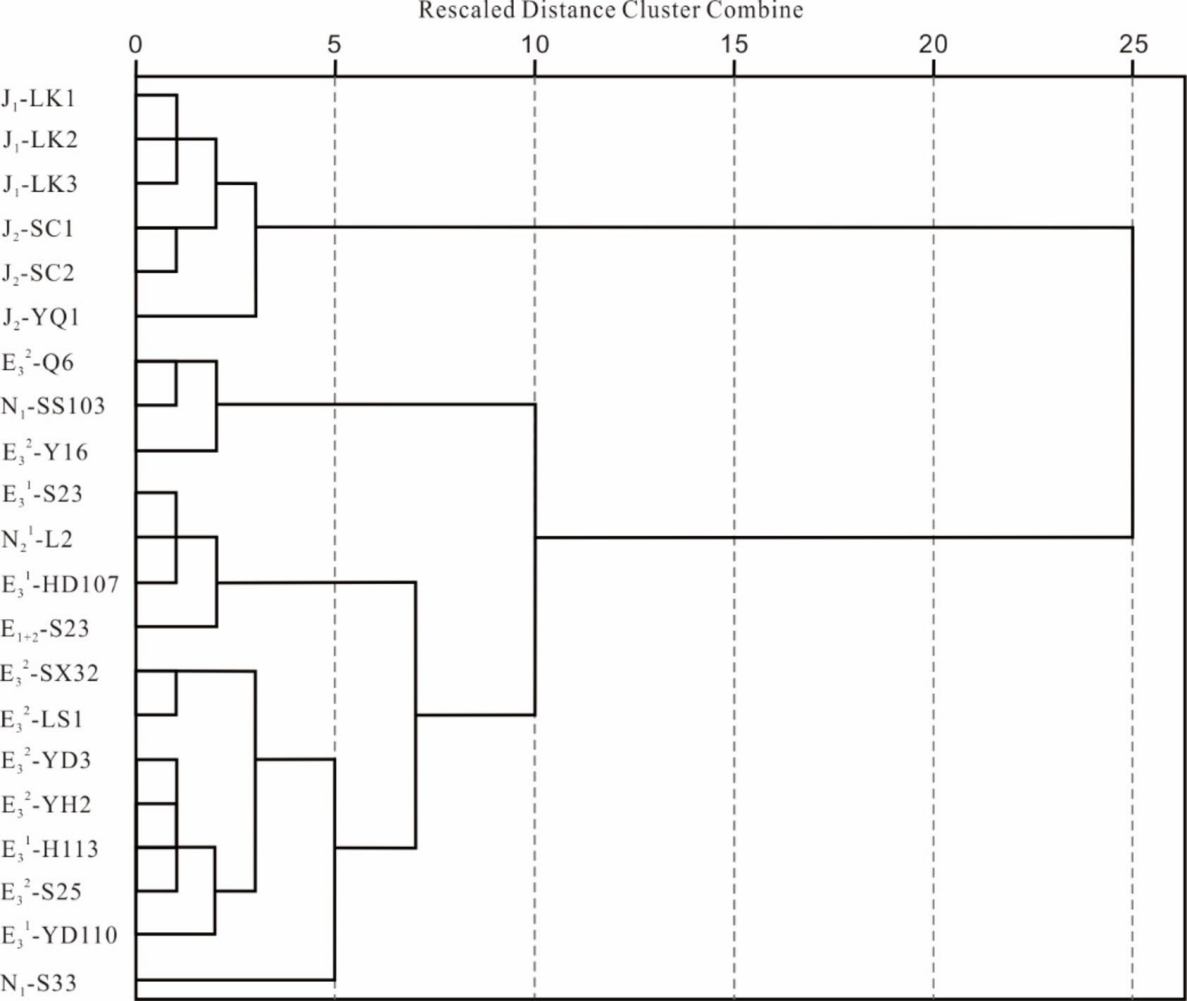
Cluster analysis dendrogram of source rocks samples in Qaidam Basin. Rescaled distances (0–25) reflect relative dissimilarity. Critical branching nodes demarcate major geochemical groups. Sample labels encode lithostratigraphic units (e.g., J_1_-LK1: the Lower Jurassic samples from Well LK1).

Based on hierarchical cluster analysis, the results are presented as follows (Fig. 7).

1. At rescaled distances < 5, samples (e.g., J_1_-LK1 and J_1_-LK2) cluster into five branches. At distances ≥ 5 and < 10, clustering at a distance of 5 merges N_1_-S33 into an adjacent branch, reducing branches to four. At distances ≥ 10 and < 15, branches decrease to two. Finally, all samples converge into one cluster at a distance of 25, reflecting significant geochemical differences between the two major branches. In fact, samples from the Paleogene and Neogene strata in the southwestern Qaidam Basin, as well as those from the Jurassic strata in the northern Qaidam Basin, were classified into two distinct groups. The C_27_ ααR/C_29_ ααR sterane ratio in the Paleogene and Neogene samples from the southwestern Qaidam Basin ranges from 0.667 to 1.723. In contrast, the Jurassic samples from the northern Qaidam Basin exhibit a narrower range, spanning from 0.512 to 0.750. This variation suggests significant in the parent materials between the two sets of source rock. Furthermore, the relative content of pregnane in the Paleogene and Neogene samples from the southwestern Qaidam Basin ranges from 0.308 to 4.512, while in the Jurassic samples from the northern Qaidam Basin, it ranges from 8.889 to 13.050. This observation indicates a higher salinity environment for the Jurassic samples in the northern Qaidam Basin. In conclusion, the geochemical characteristics of the E_3_^2^ source rock in the southwestern Qaidam Basin are markedly distinct from those of the Jurassic source rock (J_1_, J_2_) in the northern Qaidam Basin.
2. The source rock samples from E_3_^2^ in the southwestern Qaidam Basin have been categorized into two distinct groups. Samples from Well Q6 and Well Y16 were classified as the first type, characterized by the C_27_ ααR/C_29_ ααR sterane ratio ranging from 1.483 to 1.723. The second type includes all remaining samples except for the first type, with the ratio ranging from 0.667 to 1.295. This disparity in the C_27_ ααR/C_29_ ααR sterane ratio highlights significant differences between the two groups. Samples (SX32, LS1, YD3, YH2, S25) from E_3_^2^ in the southwestern Qaidam Basin show relatively distant genetic relationships with samples (HD-107, S23) from E_3_^1^, yet display a closer affinity with samples (YD110, H113) from the same E_3_^1^. Furthermore, samples (SS103) from N_1_ exhibit a close relationships with samples (Q6, Y16) from E_3_^2^, but are distant from samples (SX32, LS1, YD3, YH2, S25) from E_3_^2^. Notably, samples from N_2_^1^ and E_1+2_ do not cluster together with samples from E_3_^2^, indicating a remote genetic relationship. These results underscore the geochemical variability among source rock deposited during the same period but in different regions of E_3_^2^ in the southwestern Qaidam Basin. Moreover, the geochemical characteristics of E_3_^2^ exhibit both similarities and differences with those of N_1_ and E_3_^1^, while showing differences compared to N_2_^1^ and E_1+2_. The analysis refines the identification of these variations by systematically analyzing geochemical data, shifting from a conventional block-based approach to a well-specific evaluation in source rock assessment. This methodology establishes a robust foundation and signals the emergence of an innovative approach in hydrocarbon exploration.

**Table 3:** Biomarker parameters for typical wells of source rocks in Qaidam basin. C_23_TT/C_21_TT: C_23_ tricyclic Terpane/ C_21_ tricyclic Terpane; C_24_Te/ C_26_TT: C_24_ tricyclic Terpane/ C_26_ tricyclic Terpane; Gar/C_31_H: gammacerane/ C_31_ Hopane; C_27_/C_29_: C_27_ ααR/C_29_ ααR sterane; Preg: relative content of pregnane.

Well name	Formation	C_23_TT/C_21_TT	C_24_Te/C_26_TT	Gar/C_31_H	C_27_/C_29_	Preg (%)
S23	E_1+2_	1.740	0.449	1.605	0.667	3.309
H113	E_3_^1^	0.914	0.699	3.104	1.193	0.427
H107	E_3_^1^	0.239	1.261	1.837	0.696	0.308
S23	E_3_^1^	1.76	0.775	0.892	0.811	1.065
YD110	E_3_^1^	0.907	0.737	1.105	1.062	2.919
S25	E_3_^2^	1.816	0.494	1.248	1.239	1.641
SX32	E_3_^2^	1.717	0.276	1.687	1.295	3.068
Y16	E_3_^2^	0.874	4.481	1.352	1.483	0.952
YD3	E_3_^2^	1.777	0.464	1.354	1.071	0.539
YH2	E_3_^2^	1.449	0.796	2.339	1.124	0.771
Q6	E_3_^2^	1.164	0.548	3.074	1.723	0.688
LS1	E_3_^2^	0.959	1.866	1.126	1.264	4.512
SS103	N_1_	0.285	0.208	1.555	1.609	0.495
S33	N_1_^1^	0.636	1.558	0.461	0.941	6.926
L2	N_2_^1^	0.532	0.891	1.183	0.834	0.810
LK1	J_1_	1.104	6.157	0.053	0.535	13.052
LK 2	J_1_	0.888	8.762	0.075	0.534	13.018
LK 3	J_1_	1.088	5.912	0.071	0.629	12.819
SC1	J_2_	0.995	5.756	0.099	0.575	10.315
SC2	J_2_	0.963	7.434	0.050	0.512	11.099
YQ1	J_2_	1.013	9.603	0.126	0.750	8.889

**Table 4:** Total variance explained of biomarker parameters for typical wells of source rocks in Qaidam basin. Extraction method: principal component analysis. Cumulative variance contribution rate of pregnane (Preg) and C_27_/C_29_ sterane ratio (84.332%), exceeding the 80% threshold, indicating retained principal components capture essential data characteristics.

Component	Initial Eigenvalues			Extraction Sums of Squared Loadings		
	Total	Variance %	Cumulative %	Total	Variance %	Cumulative %
C_27_/ C_29_	3.212	64.236	64.236	3.212	64.236	64.236
Preg	1.005	20.095	84.332	1.005	20.095	84.332
Gar/C_31_H	0.418	8.357	92.689			
C_23_TT/C_21_TT	0.239	4.777	97.465			
C_24_Te/C_26_TT	0.127	2.535	100			

**Fig. 8:**
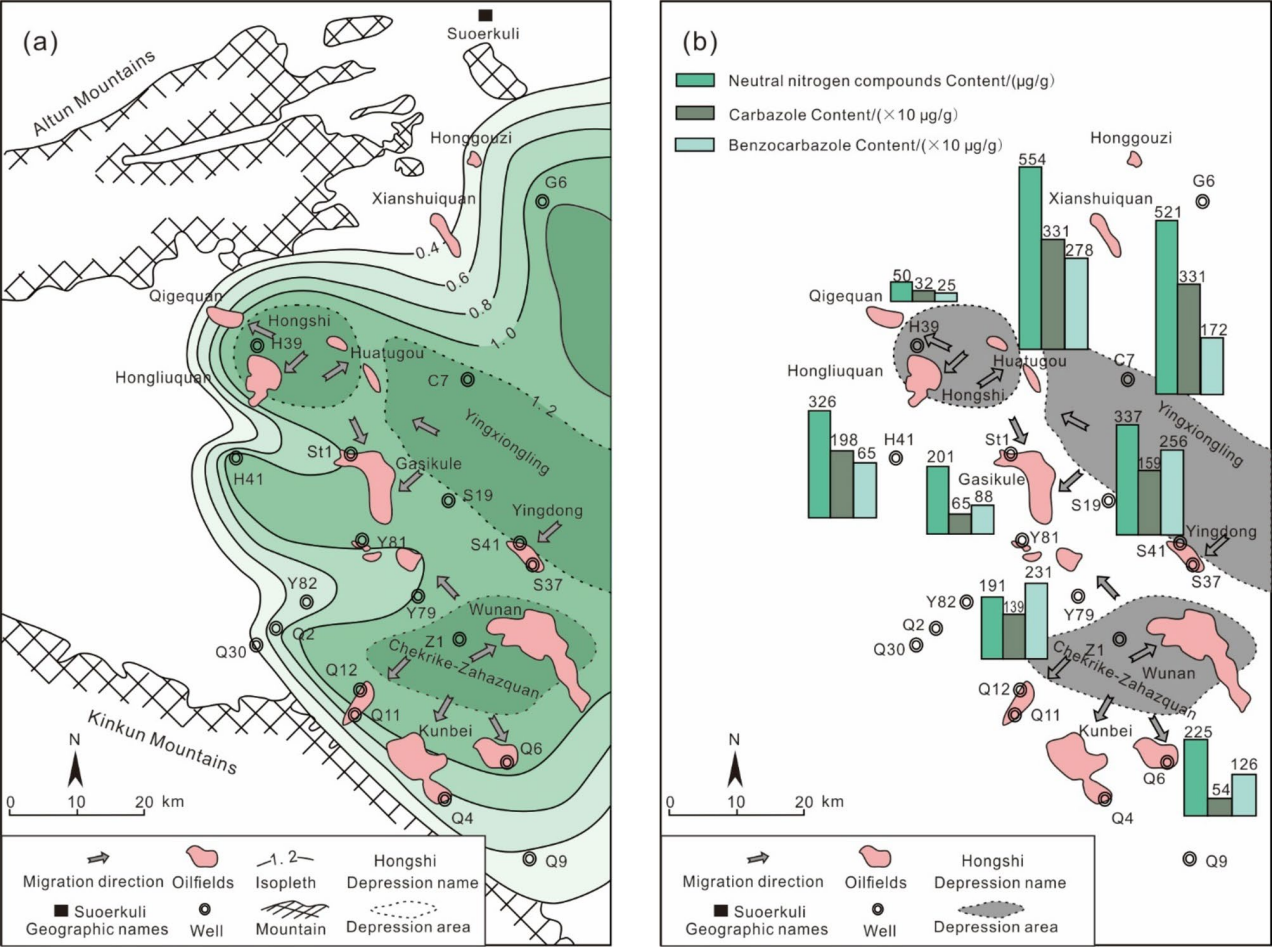
(**a**) Distribution of TOC from E_3_^2^ in the southwestern Qaidam Basin, China. (**b**) Distribution of nitrogen compounds from E_3_^2^ in the southwestern Qaidam Basin, China. The data on nitrogen compounds are cited from Reference 59. (**a**) Oil migration pathways obtained through cluster analysis. (**b**) Decreasing concentration of nitrogen-containing compounds in crude oil from the depression to the periphery, aligning with the migration pathways inferred from cluster analysis.

The E_3_^2^ source rock are widely distributed across the study area. The Hongshi, Yingxiongling, and Chekrike-Zahazquan depressions exhibit relatively high TOC levels, exceeding 1.2%, which signifies their role as prolific oil-generating regions (Fig. 8a). Following extensive exploration, seven oilfields - Qigequan, Hongliuquan, Huatugou, Gaskule, Yingdong, Wunan, and Kunbei -have been discovered in the southwestern Qaidam Basin. Analysis of crude oil samples from these fields identified two distinct types of oil, similar to the characteristics of the two types of E_3_^2^ source rock (Fig. 5). To refine the analysis of oil-source relationships, clustering analysis was employed to systematically classify and study of characteristic parameters of oil and source rock samples. The results indicate that the oils from the Qigequan and Hongliuquan oilfields are inferred to originate from the Hongshi depression, while those from Gaskule and Huatugou oilfields correlate with both the Hongshi Depression and the Yingxiongling Depression. The oil from the Yingdong oilfield is traced back Yingxiongling Depression. The Wunan and Kunbei oilfields exhibit a closer affinity to source rock in the Chekrike-Zahazquan Depression (Fig. 8a).

The Hongshi, Yingxiongling, and Chekrike-Zahazquan depressions are identified as overpressured zones, with their margins characterized by lower pressure. The spatial distribution of nitrogen-containing compounds shows a clear gradient, with the highest concentrations near the hydrocarbon-generating depressions (e.g., Yingxiongling: neutral nitrogen compounds = 554 µg/g; carbazoles = 331 × 10 µg/g; benzocarbazoles = 278 × 10 µg/g) and decreasing values toward peripheral areas (e.g., Gaskule: neutral nitrogen compounds = 201 µg/g; carbazoles = 65 × 10 µg/g; benzocarbazoles = 88 × 10 µg/g)^59^. This gradient reflects oil migration from the depression centers to their margins, indicating that the crude oil originates from adjacent source rocks (Fig. 8b). A significant deviation is observed in the Hongliuquan Oilfield, where the concentrations of nitrogen compounds are significantly higher than in the surrounding areas. This is likely due to the connection between the Hongliuquan Oilfield and the Yingxiongling depression by the Shizigou-Youshashan fault, which shortens the migration distance and reduces the adsorption of nitrogen compounds. The elevated levels of these compounds in the Hongliuquan Oilfield imply a potential contribution from both the Hongshi and Yingxiongling Depressions.

The migration pathways inferred from nitrogen-containing compound analysis support the oil-source correlation results obtained through cluster analysis, demonstrating the reliability of the latter method. Cluster analysis is expected to complement to nitrogen compounds and isotopic analyses in analyzing hydrocarbon migration pathways. Moreover, it will play a critical role in advancing understanding of hydrocarbon accumulation mechanisms and guiding future petroleum exploration strategies.

## Conclusions

1. The threshold TOC value for the source rock in the Upper part of the Lower Ganchaigou Formation in the southwestern Qaidam Basin is 0.4%, as determined by its positive correlation with chloroform bitumen ‘A’ and the Pg. The analysis of the samples indicates that the E_3_^2^ source rock exhibit fair hydrocarbon generation potential.
2. Pyrolysis and hydrocarbon group composition reveal a complex mixture of organic matter types within the source rock, predominantly composed of Type I and Type II_1_ kerogens. The organic matter is derived from both aquatic organisms and higher plants, with a greater contribution from aquatic organisms. The depositional environments of organic matter are characterized by either highly reducing and saline conditions or relatively weak reducing and low-salinity conditions. According to assessment using Ro and T_max_ parameters, the organic matter is currently classified as low-maturity to mature.
3. Cluster analysis was used to compare the geochemical characteristics of source rock in different geological periods. The results highlight the diversity in geochemical attributes among source rock deposited contemporaneously but in different regions of the E_3_^2^ in the southwestern Qaidam Basin. Moreover, the geochemical properties of E_3_^2^ exhibit both similarities and differences compared to those of N_1_ and E_3_^1^, yet they display pronounced differences in comparison with N_2_^1^, E_1+2_, J_1_ and J_2_.
4. The crude oils from Qigequan and Hongliuquan oilfields are inferred to originate from the Hongshi depression, while the crude oil from the Gaskule oilfield is associated with source rocks from both the Hongshi Depression and the Yingxiongling depression. Meanwhile, the Wunan and Kunbei oilfields exhibit a stronger association with source rocks located in the Chekrike-Zahazquan depression. These insights have guided subsequent hydrocarbon exploration strategies, providing a novel framework for evaluating origins of mixed-source crude oils and delineating hydrocarbon migration pathways.

## Data availability

The datasets used and/or analyzed during the current study available from the corresponding author on reasonable request.

## References

[1] Yasin, Q., Baklouti, S., Sohail, G. M., Asif, M. & Xufei, G. Evaluation of neoproterozoic source rock potential in SE Pakistan and adjacent Bikaner–Nagaur basin India. *Sci. Rep.* **12**, 11102. 10.1038/s41598-022-14831-5 (2022). 35773280 10.1038/s41598-022-14831-5 PMC9247107

[2] Diab, A. I., Sanuade, O. & Radwan, A. E. An integrated source rock potential, sequence stratigraphy, and petroleum geology of (Agbada-Akata) sediment succession, Niger delta: application of well logs aided by 3D seismic and basin modeling. *J. Pet. Explor. Prod. Technol.* **13**, 237–257. 10.1007/s13202-022-01548-4 (2023).

[3] Agbor, F. A., Mhlambi, S. & Teumahji, N. A. M. Petroleum source rock potential evaluation: a case study of block 11a, pletmos sub-basin, offshore South Africa. *J. Pet. Explor. Prod. Technol.* **13**, 995–1007. 10.1007/s13202-022-01599-7 (2023). Sonibare, W. A., Van Bever Donke, J. .

[4] Garba, T. E. & Mustapha, K. A. Source rock characterisation and petroleum system modelling: a review of marginal marine deposit in the Permo-Triassic Sydney basin, Australia. *J. Sediment. Environ.* **9**, 239–251. 10.1007/s43217-024-00168-8 (2024).

[5] Wang, G. C., Sun, M. Z., Gao, S. F. & Tang, L. The origin, type and hydrocarbon generation potential of organic matter in a marine-continental transitional facies shale succession (Qaidam basin, China). *Sci. Rep.* **8**, 6568. 10.1038/s41598-018-25051-1 (2018). 29700353 10.1038/s41598-018-25051-1 PMC5920063

[6] Salman, S. A., El-Anwar, A., Makled, E. A., Mahfouz, W. A., Belal, Z. L. & K. H. & Hydrocarbon generation potential evaluation via petrographic and geochemical analyses of El-Maghara coal in Sinai, Egypt. *Sci. Rep.* **14**, 860. 10.1038/s41598-024-51291-5 (2024). 38195973 10.1038/s41598-024-51291-5 PMC10776611

[7] Shalaby, M. R., Jumat, N., Lai, D., Malik, O. & Integrated TOC prediction and source rock characterization using machine learning, well logs and geochemical analysis: case study from the jurassic source rocks in Shams field, NW desert Egypt. *J. Pet. Sci. Eng.* **176**, 369–380. 10.1016/j.petrol.2019.01.055 (2019).

[8] Guo, Y. et al. An integrated organic–inorganic geochemical characterization of paleogene sediments in 1 structural belt of the Nanpu Sag, Bohai Bay basin, Eastern China: implications for the origin of organic matter. Geochem. *Explor. Environ. Anal.* **21**, geochem2019–geochem2060. 10.1144/geochem2019-060 (2020).

[9] Huang, D. F., Li, J. C. & Zhang, D. J. The types of kerogen and the effectiveness, limitations, and relevance of classification parameters. *Acta Sedimentol. Sin*. **2**, 18–35 (1984).

[10] Jiang, D. X. & Yang, H. Q. Palynological evidence for the tertiary petroleum source of the Qaidam basin. *Acta Bot. Sin*. **40**, 77–82 (1998).

[11] Meng, B. K. et al. Distribution characteristics and significance of the aromatic hydrocarbons molecular biomarker in crude oil from the Northwestern Qaidam basin. *Nat. Gas Geosci.* **32**, 738–753 (2021).

[12] Peng, D. H., Su, A. G., Zhu, Y. M., Guo, J. & Zhang, B. Q. Evolutionary process of hydrocarbon generation and characteristics of sourcerocks of the tertiary salt lacustrine facies in the West of Qaidam basin. *Acta Pet. Sin*. **26**, 92–101 (2005).

[13] Zhang, B., He, Y. Y., Chen, Y., Men, Q. Y. & Yuan, L. Geochemical characteristics and oil accumulation significance of the high-quality saline lacustrine source rocks in the Western Qaidam basin, NW China. *Acta Pet. Sin*. **38**, 1158–1167. 10.7623/syxb201710006 (2017).

[14] Zhu, Y. M. et al. Geochemical characteristics of tertiary saline lacustrine oils in the Qaidam basin. *Chin. J. Geol.* **39**, 475–485 (2004).

[15] Tian, X. Y. et al. A further discussion on source control theory—by the example of marine carbonate gas reservoir in the Sichuan basin. *Acta Geol. Sin*. **40**, 410–415. 10.3969/j.issn.1006-0995.2020.03.013 (2020).

[16] Yang, Z. & Zou, C. N. Exploring petroleum inside source kitchen: connotation and prospects of source rock oil and gas. *Pet. Explor. Dev.* **46**, 173–184 (2019).

[17] Zhang, L. et al. High-quality oil-prone source rocks in Jiyang Depression. *Geochim*, 32, 35–42, DOI: https://doi.org/10. 19700/ j. 0379–1726. 01. 005 (2003). (2003).

[18] Zhang, L. Y. A restudy on the hydrocarbon occurrence of enriched organic matter: A case study of Dongying depression. *Geochim* **34**, 619–625. 10.19700/j.0379-1726.2005.06.009 (2005).

[19] Lu, S. F. et al. Evaluation criteria of high-quality source rocks and its applications: taking the Wuerxun Sag in Hailaer basin as an example. *Earth Science—J China Univ. Geosci.* **37**, 535–544. 10.3799/dqkx.2012.060 (2012).

[20] Zhang, W. Z., Yang, H., Li, J. F. & Ma, J. Leading effect of high-class source rock of Chang 7 in Ordos basin on enrichment of low permeability oil-gas accumulatio—hydrocarbon generation and expulsion mechanism. *Pet. Explor. Dev.* **33**, 289–293 (2006).

[21] Fu, S. T. Key controlling factors of oil and gas accumulation in the Western Qaidam basin and its implications for favorable exploratjon direction. *Acta Sedimentol. Sin*. **28**, 373–379. 10.7623/syxb2016s1001 (2010).

[22] Li, H. B., Zhang, M., Zhang, C. M. & Peng, D. H. Evaluation of tertiary source rocks in the Western Southern area of Qaidam basin. *J. Oil Gas Technol.* **28**, 41–43 (2006).

[23] Zhao, Y. et al. Oil-source analysis for Chang-9 subsection (Upper Triassic) of Eastern Gansu Province in Ordos basin. *Acta Sedimentol. Sin*. **33**, 1023–1032. 10.14027/j.cnki.cjxb.2015.05.018 (2015).

[24] Peters, K. E. & Moldowan, J. M. *The Biomarker Guide: Interpreting Molecular Fossils in Petroleum and Ancient Sediments*1–363 (Cambridge University Press, 1993).

[25] Wang, X., Yang, H., Kang, S. F., Zhang, Y. Q. & Jia, X. Y. Analysis on oil sources and reservoir formation of well Lu 9 in Luliang uplift in Junggar basin. *Xinjiang Pet. Geol.* **22**, 213–216 (2001).

[26] Jiang, W., Ablimit, I., LI, H., Chen, J. & Li, Z. H. Geochemical characteristics and identification of mixed crude oil of the Baikouquan formation lower Wuerhe formation on the East slope of the Mahu Sag, Junggar basin. *Geochim* **50**, 185–198. 10.19700/j.0379-1726.2021.02.005 (2021).

[27] Peters, K. E., Ramos, L. S., Zumberge, J. E., Valin, Z. C. & Bird, K. J. De-convoluting mixed crude oil in prudhoe Bay field, North slope, Alaska. *Org. Geochem.* **39**, 623–645. 10.1016/j.orggeochem.2008.03.001 (2008).

[28] Alizadeh, B., Alipour, M., Chehrazi, A. & Mirzaie, S. Chemometric classification and geochemistry of oils in the Iranian sector of the Southern Persian Gulf basin. *Org. Geochem.* **111**, 67–81. 10.1016/j.orggeochem.2017.05.006 (2017).

[29] Wu, N., Liu, X. F. & Xu, T. Study on the trace of Hydmcarbon migration path. *Spec. Oil Gas Reserv.* **14**, 28–32 (2007).

[30] Zhang, Y. S. et al. Genesis, type, and reservoir formation law of natural gas in Western Qaidam basin. *China Pet. Explor.* **24**, 498–508. 10.3969/j.issn.1672-7703.2019.04.010 (2019).

[31] Duan, Y. et al. Geochemical characteristics of hydrocarbons in crude oils from the Qaidam basin. *Pet. Geol. Exp.* **26**, 359–364 (2004).

[32] Feng, Z. H. et al. Depositional environment of terrestrial petroleum source rocks and geochemical indicators in the Songliao basin. *Sci. China Earth Sci.* **54**, 1304–1317. 10.1007/s11430-011-4268- (2011).

[33] Lu, S. F. et al. Evaluation criteria of high-quality source rocks and its applications: taking the Wuerxun Sag in Hailaer basin as an example. *Earth Sci. J. China Univ. Geosci.* **37**, 535–544. 10.3799/dqkx.2012.060 (2012).

[34] Peng, D. H., Su, A. G., Zhu, Y. M., Zhang, B. Q. & Guo, J. Evolutionary process of hydrocarbon generation and characteristies of sourcerocks of the tertiary salt lacustrine facies in the West of Qaidam basin. *Acta Pet. Sin*. **26**, 92–96 (2005).

[35] Zhang, C. H. et al. Characteristics of source rocks in the upper member of lower Ganchaigou formation in Ganchaigou area, Qaidam basin. *Mud Logging Eng.* **34**, 139–144. 10.3969/j.issn.1672-9803.2023.02.022 (2023).

[36] Behar, F., Beaumont, V. I., Penteado, H. L. & De, B. Rock-Eval 6 technology: performances and developments. *Oil Gas Sci. Technol.* **56**, 111–134. 10.2516/ogst:2001013 (2001).

[37] Romero-Sarmiento, M. F., Ramiro-Ramirez, S., Berthe, G., Fleury, M. & Littke, R. Geochemical and petrophysical source rock characterization of the Vaca muerta formation, Argentina: implications for unconventional petroleum resource estimations. *Int. J. Coal Geol.* **184**, 27–41. 10.1016/j.coal.2017.11.004 (2017).

[38] Romero-Sarmiento et al. New Rock-Eval method for characterization of shale plays. In: 14th Latin American Congress on Organic Geochemistry (ALAGO), Buzios, Rio de Janeiro – Brazil (2014).

[39] Romero-Sarmiento, M. F., Euzen, T., Rohais, S., Jiang, C. & Littke, R. Artificial thermal maturation of source rocks at different thermal maturity levels: application to the triassic Montney and Doig formations in the Western Canada sedimentary basin. *Org. Geochem.* **97**, 148–162, 10.1016/j.orggeochem.2016.05.002 (2016a).

[40] Romero-Sarmiento et al. New Rock-Eval method for characterization of unconventional shale resource systems. *Oil Gas Sci. Technol.* **71**, 37. 10.2516/ogst/2015007 (2016b).

[41] Xiong, D. M., Ma, W. Y., Zhang, M. F., Wu, C. J. & Tuo, J. C. New method for the determination of kerogen type and the hydrocarbon potential. *Nat. Gas Geosci.* **25**, 898–905. 10.11764/j.issn.1672-1926.2014.06.0898 (2014).

[42] Tissot, B. & Durand, B. Influence of natures and diagenesis of organic matter in the formation of petroleum. *AAPG Bull.* **58**, 438–459. 10.1306/83D91425-16C7-11D7-8645000102C1865D (1974).

[43] Yang, W. L., Li, Y. K., Gao, R. Q. & Guo, Q. F. Types and evolutionary patterns of continental source rocks for oil generation in the Songliao basin. *Sci. China Earth Sci.* **8**, 1000–1008 (1981).

[44] Welte, D. H. & Tissot, B. P. *Petroleum Formation and Occurrence* 2nd edn 131–135 (Springer, 1984). 10.1007/978-3-642-87813-8

[45] Duan, Y., Zhang, S. B., Zheng, C. Y. & Wu, B. X. Study on the genesis of crude oil in the Yan’an formation of the Maling oilfield, Ordos basin. *Acta Geol. Sin*. **81**, 1407–1415 (2007).

[46] Zhang, D. W. Research progress on oil and gas geology and exploration practice in Qaidam basin. *Xinjiang Pet. Geol.* **40**, 505–512. 10.7657/XJPG20190501 (2019).

[47] Hao, W. X. Yangtze University,. Study on the organic Geochemical characteristics and formation mechanism of source rocks in the upper member of the Xiaganchaigou Formation, Yingxiong Ling area, Chaixi Depression (ed. Hao W. X.) 27–34 (2023).

[48] Moldowan, J. M., Seifert, W. K. & Gallegos, E. J. Relationship between petroleum composition and the depositional environment of petroleum source rocks. *AAPG Bull.* **69**, 1255–1268. 10.1306/AD462BC8-16F7-11D7-8645000102C1865D (1985).

[49] Peters, K. E., Walters, C. C. & Moldowan, J. M. *The Biomarker Guide* (2nd ed). 72–80, (2005). 10.1017/CBO9781107326040 (United Kingdom at the tniversity Press.

[50] Sheng, G. Y., Lu, H., Liao, J. & Peng, P. A. Advances in novel hopanoids present in geological bodies. *Geochim* **48**, 421–446. 10.19700/j.0379-1726.2019.05.001 (2019).

[51] Sun, P., Tang, Y. J., Zhang, Y. S. & Peng, Y. The characteristics of biomarker compounds of source rocks in Linxi formation in guandi section of Linxi County in the East of inner Mongolia. *Acta Pet. Sin*. **13**, 1–7. 10.16772/j.cnki.1673-1409.2016.32.001 (2016).

[52] Zhang, M. F. et al. Geochemical characteristics of the source rocks from Wunan-Lücaotan area in Qaidam basin. *Nat. Gas Geosci.* **23**, 636–645 (2012).

[53] Zhang, H. et al. Dominant lithofacies and factors controlling reservoir formation of the shale sequence in the upper member of the paleogene lower Ganchaigou formation, Ganchaigou area, Qaidam basin. *Oil Gas Geol.* **45**, 1305–1320. 10.11743/ogg20240508( (2024).

[54] Chen, Z. J. et al. Characteristics and geochemical indication of Over-Mature source rocks in the paleozoic, Yingen-Ejinaqi basin. *Oil Gas Geol.* **43**, 682–695. 10.11743/ogg20220316 (2022).

[55] Radke, M. & Welte, D. H. The Methylphenanthrene index (MPI): A maturity parameter based on aromatic hydrocarbons. Advances in Organic Geochemistry. Chichester: John Wiley and Sons Incorporation, 504–512 (1981).

[56] Luo, B. J. & Li, X. Y. Characteristics of aromatic hydrocarbons in crude oils. *Chin. J. Geochem.* **13**, 97–106. 10.1007/BF02838510 (1994).

[57] Zhu, Y. M. Thermal evolution and maturity parameters of pentacyclic aromatic hydrocarbons in source rocks. *Geol. Geochem.* **1**, 75–80 (1998).

[58] Chen, Y. et al. Relationship between the Methylphenanthrene index Methylphenanthrene ratio and organic thermal evolution: take the Northern margin of Qaidam basin as an example. *Pet. Explor. Dev.* **37**, 508–512 (2010).

[59] Guo, Z. Q., Zhong, J. H., Liu, W. H., Rao, M. Y. & Liu, Y. T. Relationship between abnormal overpressure and reservoir formation in the tertiary of the Western Qaidam basin. *Acta Pet. Sin*. **25**, 13–18 (2004).

